# Prognosis of Implants with Implant-Supported Fixed Dental Prostheses in the Elderly Population: A Retrospective Study with a 5- to 10-Year Follow-Up

**DOI:** 10.3390/healthcare10071250

**Published:** 2022-07-04

**Authors:** Tomoyo Takahashi, Masafumi Kihara, Kyosuke Oki, Tatsuya Matsuzaki, Yasunori Ayukawa, Yasuyuki Matsushita, Kiyoshi Koyano

**Affiliations:** 1 Section of Implant and Rehabilitative Dentistry, Division of Oral Rehabilitation, Faculty of Dental Science, Kyushu University, Fukuoka 812-8582, Japan; tomoyo-n@dent.kyushu-u.ac.jp (T.T.); ayukawa@dent.kyushu-u.ac.jp (Y.A.); matsushi@dent.kyushu-u.ac.jp (Y.M.); 2 Section of Fixed Prosthodontics, Division of Oral Rehabilitation, Faculty of Dental Science, Kyushu University, Fukuoka 812-8582, Japan; o-ki@dent.kyushu-u.ac.jp (K.O.); tmatsu@dent.kyushu-u.ac.jp (T.M.); 3 Division of Advanced Dental Devices and Therapeutics, Faculty of Dental Science, Kyushu University, Fukuoka 812-8582, Japan; koyano@dent.kyushu-u.ac.jp

**Keywords:** elderly population, implant-supported fixed dental prostheses (ISFDPs), prosthetic intervention, survival rate

## Abstract

This retrospective study aimed to investigate the survival rate of implants from 5 to 10 years after the placement of implant-supported fixed dental prostheses (ISFDPs) and the management of implant loss in the elderly population. Elderly patients (≥65 years old) who had been treated with ISFDPs and followed up with for at least 5 years between October 2009 and March 2020 were enrolled. Patient profiles and implant-related data were extracted. The survival rate of implants up to 5 years as well as the 10-year cumulative survival rate were evaluated. The management of implant loss and prosthetic interventions were also investigated. In total, 195 patients (mean age: 70.1 ± 4.5 years old) and 687 implants (287 ISFDPs) were assessed. The 5-year survival rate was 99.0% and the 10-year cumulative survival rate was 98.1%. Seven of the eleven implants lost were lost due to peri-implantitis. Only three implants in two patients were placed after the loss of the implants; most were restored using non-invasive procedures. Two patients underwent a conversion from ISFDPs to removable prostheses. This study showed that high survival rates were observed in an elderly population with ISFDPs and that non-invasive procedures were often applied after the loss of an implant.

## 1. Introduction

The proportion of persons aged 65 years old or older is increasing in Japan, and Japan is already a super-aged society, with more than 21% of the population being 65 years old or older [1]. The increasing demand for dental interventions for edentulous or partially edentulous patients has been noted [2,3]. Implants are regarded as one of the main treatment modalities for the elderly population with partially or completely edentulous arches [4,5]. In general, previous reports have proven that dental implant treatments for partially or completely edentulous patients could be a solution to declined oral function and poor aesthetics [6,7,8,9,10]. Although aging exerts a certain effect on systemic and oral health [11,12], previous studies have shown that aging alone does not affect implant treatment and its prognosis [13,14,15,16]. However, aging-associated factors, such as systemic diseases, and peri-implant tissue inflammation due to a poor oral hygiene capacity might be risk factors affecting the longevity of implants [17]. All patients have potential risks regarding difficulty attending maintenance appointments for their implants and prostheses, depending on their age. Previous studies have also suggested the need for effective management for future maintenance [18,19,20]. This includes the improvement and simplification of the intraoral condition. In addition, oral care carried out by the patients and caregivers must be simple. However, these conditions depend on their oral environments. Dentists and dental hygienists need to know the oral condition of elderly patients with implant-supported prostheses.

Implant-related complications have been categorized into two main types: biological and technical complications [21,22]. Although previous systematic reviews have reported that these complications can be risk factors for implant loss [23,24,25,26,27,28], prosthetic management after implant loss, especially in the elderly population, has not been reported on proactively. The reason for implant loss must be understood in order to discuss how to manage these complications in the elderly population for future maintenance in a longitudinal study.

This retrospective study investigated the survival rate of implants in the elderly population after the use of implant-supported fixed dental prostheses (ISFDPs) in a university hospital. In addition, prosthodontic management after implant loss were investigated to inform the discussion on the management of ISFDPs in the elderly population. The hypothesis of this study was that implant treatments with ISFDPs for the elderly population could have a high success rate, and management after the loss of implants was also considered successfully.

## 2. Materials and Methods

### 2.1. Ethics

Ethics approval for the present study was obtained from our institutional ethical review board (#2021-449). The institutional review board confirmed that the requirement to obtain informed consent was waived due to this being a retrospective observational study. This observational study was conducted according to the guidelines of Strengthening the reporting of observational studies in epidemiology (STROBE). All patients included in this study provided informed consent prior to implant treatment.

### 2.2. Study Subjects

This retrospective study aimed to analyze elderly patients (≥65 years old) who had been treated with ISFDPs and were followed up with between October 2009 and March 2020 in Kyushu University Hospital. This study was designed as a longitudinal retrospective study, and patients who had been followed for at least 5 years after the placement of definitive ISFDPs were enrolled in this study. The patients who had been treated with implant-supported removable prostheses, such as implant overdentures (IODs) or implant-assisted removable partial dentures (IARPDs), were excluded from the study. Patients who experienced implant loss within 5 years were included in the data analyses.

### 2.3. Data Collection and Analyses

Data collected from the patients’ medical charts retrospectively were used for the analyses. The following items were extracted; age at the placement of the ISFDP (baseline), gender, number of implants, number of units of the ISFDP (single, 2-unit by two implants, or ≥3-unit by two or more implants), implant position (number of units and anterior: incisors and canine, posterior: premolars and molars, or combination), jaws (maxilla or mandible), retention type (screw: SC or cement: CM), and materials of the definitive ISFDPs (metal: M, metal–resin: MR, metal–ceramic: MC, all ceramic (other than monolithic zirconia): AC, or monolithic zirconia: MnZ). The functioning periods, including the loss of implants, were also extracted. In cases of lost implants, the functioning periods were defined as the periods from the placement of the definitive ISFDPs to the time of implant loss.

The survival rate of implants up to 5 years was calculated. The 10-year cumulative survival rate was also evaluated with Kaplan–Meier curves. The interventions used after the loss of implants were also evaluated. In addition, we extracted the patients whose prosthetic designs were changed for any reason.

## 3. Results

### 3.1. Study Subjects and Implants

The retrospective medical chart review showed that a total of 1220 patients received 3808 implants and 1866 prostheses from October 2009 to March 2020, and 195 of them, 76 males and 119 females with a total of 687 implants and 287 prostheses, were included in the study based on the inclusion and exclusion criteria (Table 1). The mean age was 70.1 ± 4.5 years old (male: 69.9 ± 4.7, female: 70.2 ± 4.4 years old). The total number of implants and ISFDPs in these patients was 687 (maxilla: 339, mandible: 348) and 287 (maxilla: 133, mandible: 154), respectively. Detailed information on the implants is given in Table 2 and Table 3.

### 3.2. Survival Rates and Detailed Information for the Lost Implants and Their Management

**Figure 1:**
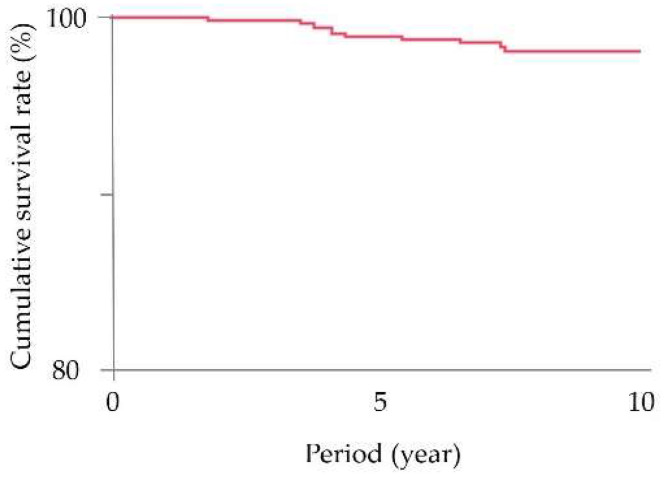
Kaplan–Meier curves of implants in elderly patients with implant-supported fixed dental prostheses.

The survival rate of implants after the delivery of ISFDPs was shown using Kaplan–Meier curves (Figure 1).

In the first 5 years, seven implants in six patients were lost, and the 5-year survival rate was 99.0%. Additional lost implants from 5 years to 10 years included four implants in four patients, and the 10-year cumulative survival rate was 98.1% (Table 4).

Detailed information on the lost implants is shown in Table 5. In the first 5 years, all the lost implants had been placed in molar sites and were screw-retained. No single unit implants were lost. The materials of the suprastructures in the lost implants included four metal–resin prostheses (4/122:3.3%) and three metal–ceramic prostheses (3/133:2.3%). One implant was removed due to the patient’s discomfort. This patient was rehabilitated with an IARPD using the remaining implant. Six implants in five patients were lost due to peri-implantitis. After the loss of the implants, two patients underwent implant placement again. One of them had two more implants adjacent to the lost site (lost: 46, and new implants: 44 and 45) and the other chose a removable partial denture (RPD) at first but later chose to have an implant at the lost site. An RPD was placed in one patient. Two patients underwent modification of their suprastructures. From 5 years up to 10 years, all the lost implants had been placed in molars (two implants) and premolars (two implants) and were screw-retained. The materials of the suprastructures in the lost implants included three metal–resin prostheses (3/122:2.5%) and one metal–ceramic prosthesis (1/133:0.8%). Two implants were lost due to peri-implantitis. One implant was lost in connection with bone resection due to gingival cancer and the other one was lost due to bone resorption without remarkable inflammation. After the loss of the implants, one patient was rehabilitated with IARPD, and an RPD was placed for one patient. Two patients were managed with the modification of the suprastructure.

### 3.3. Conversion of the Suprastructure from a Fixed to Removable Prosthesis without Implant Loss

**Figure 2:**
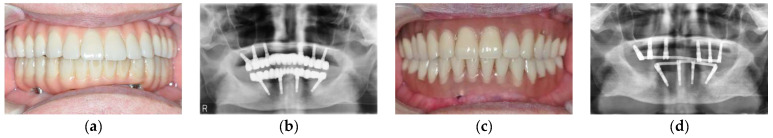
A case of suprastructure conversion (86 years old at baseline, male): (**a**) intraoral view with fixed prostheses; (**b**) panoramic image with fixed prostheses; (**c**) intraoral view with removable prostheses (implant overdentures); (**d**) panoramic image with bar attachments for implant overdentures.

**Figure 3:**
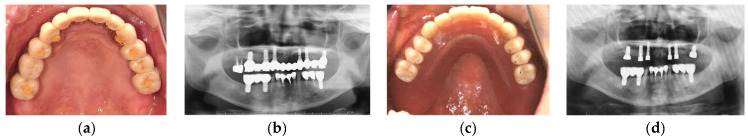
A case of suprastructure conversion (70 years old at baseline, female): (**a**) occlusal view with fixed prosthesis; (**b**) panoramic image with fixed prostheses; (**c**) occlusal view with removable prosthesis (implant overdenture); (**d**) panoramic image with magnet attachments for implant overdenture.

A conversion of the suprastructure without implant loss was conducted in only two patients. One patient (86 years old at baseline, male) was rehabilitated with a complete ISFDP in the maxilla. He was also treated with a complete ISFDP in the mandible prior to the inclusion period. Both prostheses were changed into IODs. The IODs were fabricated due to the caregiver’s suggestion (difficulty of cleaning) (Figure 2). The other female patient was 70 years old at baseline. Her left and right molars in the mandible were restored with ISFDPs during the inclusion period. A maxillary complete ISFDP had been placed prior to the inclusion period. This prosthesis was changed into an IOD because of the mucosal inflammation beneath the prosthesis. Alterations to IOD improved their chief complaints, resulting in better cleaning and inflammation extinction (Figure 3).

## 4. Discussion

This study clearly showed the high survival rate of implants in the elderly population after the delivery of ISFDPs after 5 years and up to 10 years. This study evaluated the survival rate of implants after the use of ISFDPs, meaning that all implants included in this study had achieved osseointegration successfully. These findings support previous studies that suggested that ISFDPs are a predictable long-term treatment option in the elderly population [13,14,15,16]. Eight of the eleven lost implants were due to peri-implantitis and one implant was lost due to bone resorption without inflammation. These implants were diagnosed with no prospect of recovery and were removed under the informed consent of patients. One implant was lost with resection of the bone due to gingival cancer. In total, ten implants were lost due to biological complications and one implant was removed due to a psychological reason (the patient claimed discomfort but no medical problem was indicated) without any other complications such as peri-implantitis, implant fracture, and so on. This retrospective analysis showed that biological complications, including peri-implant mucositis, peri-implantitis, and bone resorption, occurred in at least 23 patients in 5 years and 16 patients in 10 years (these complications were extracted from medical charts; some complications, especially slight or mild peri-implant mucositis, might not have been recorded in the medical chart). No implants were lost due to technical complications such as implant fracture, although technical complications were identified in at least 18 patients after 5 years and 6 patients after 10 years (abutment screw loosening or the wear/chipping of the veneering materials. Similar to the biological complications, some of these complications, especially minor ones, might not have been recorded in the medical chart).

Peri-implantitis can be attributed to plaque accumulation. Poor plaque control skills and a lack of regular maintenance care after the placement of prostheses increase the morbidity risk [29]. Previous studies showed the wide range of prevalence of peri-implantitis [30,31,32]. The importance of regular maintenance to prevent biological complications has been reported [33,34]. The subjects in this study were followed up with for at least 5 years, and this suggested that regular maintenance by dentists and/or dental hygienists contributed to the high survival rate. However, the prognosis of the patients who were not followed up with professionals should be of concern, and maintenance by other dentists should be requested to avoid severe issues as much as possible, along with providing some information about implant treatment [18]. In regard to chemical plaque control in the present study, although chlorhexidine (CHX) has been the most popular and is proven to be an effective oral rinse [35], we did not use CHX for this purpose because the use of CHX on mucosal surfaces including the oral cavity has not obtained pharmaceutical approval in Japan. The use of CHX on our patients has a possibility to reduce the chance of peri-implantitis.

In the present study, all the lost implants were a submerged type with a butt-joint connection between the implant body and abutment. This had some possibility to increase the chance of peri-implantitis in the present study. Due to the presence of a microgap, microleakage can occur at the implant–abutment connection of this type [36] and it leads to the accumulation of bacteria and subsequent inflammation and bone resorption. In contrast, a non-submerged-type implant does not have a microgap near the bone, and a submerged-type implant with a platform-shifting and conical connection between the implant body and abutment can reduce the risk of microleakage [36]. The use of a non-submerge type or a submerged type with a conical connection implant may reduce the risk of peri-implantitis.

In addition, one implant was lost due to bone resorption without definite inflammation. Although a correlation between occlusal overload or occlusal trauma and peri-implant bone loss has been suggested, this correlation has only been rarely reported, and little evidence has been provided to support a cause-and-effect relationship in humans [37,38]. More specific research and findings are required. Even if occlusal inappropriateness is not necessarily correlated with implant failure, a significant relationship has been reported between the distribution of occlusal contacts and temporomandibular disorder [39]. Occlusal contact should be checked at every check-up.

One implant was removed with resection of the bone due to gingival cancer. In this case, the implant was placed in the bone and was scheduled to be resected. However, chemotherapy and radiotherapy in the craniofacial area has an adverse effect on the prognosis of an implant placed even at the distant site to tumor [40]. In such cases, the prevention of peri-implant mucositis and subsequent peri-implantitis should be considered. Granulocyte-macrophage colony-stimulating factor therapy is a possible candidate to prevent and treat mucositis [41].

Two of the ten patients underwent implant placement again after the loss of the implant. One patient had received implants at mandibular molar sites bilaterally. One implant was lost during the observation period and an additional implant was placed. The other patient had RPD just after the loss of the implant and subsequently received two implants. He was rehabilitated with two ISFDPs in the mandible. However, further follow-up was impossible because of ill health. As mentioned above, offering information on implant treatment should be considered.

Management using IARPD in two patients and the modification of the suprastructure in four patients were identified. Both management strategies were non-invasive procedures and cost-efficient. Although the effects of IARPD and a shortened dental arch (SDA) on oral health-related quality of life were reported [42,43], few studies have evaluated the treatment effects after conversion from ISFDP. However, in the elderly population, less invasive procedures should be considered depending on the systemic conditions of the patient, and simplifying the oral environment and oral care is recommended for future maintenance [19,20]. In addition, a patient who lost a single unit implant was also rehabilitated with RPD. Future studies will be required to assess the effect of the alteration of the prosthesis, especially from fixed to removable, on the function or the patient’s satisfaction after any trouble with their implants.

Two patients experienced the conversion of their prostheses from fixed to removable ones. Both patients showed inflammation of the mucosa beneath a full arch ISFDP. The conversion to a removable prosthesis clearly improved the mucosal condition. A previous study showed that the ease of cleaning was significantly better with removable prostheses compared to fixed ones [44]. This procedure might lead to the simplification of prostheses and oral care, which has been recommended for future maintenance [20]. However, more detailed information, such as a functional and psychological assessment, should be obtained and compared with a sufficient number of subjects.

The limitations of present study are as follows. First, in the present study, all subject which fulfilled the inclusion criteria (and did not meet the exclusion criteria) were enrolled and general health status was not considered. Diabetes or other general health conditions may have a strong impact on the prognosis of the implant. Next, since this study was conducted in a retrospective manner and the subjects were collected only from one general hospital, there may be a bias in the enrolled subjects. Especially, subjects were limited to outpatients who could attend by themselves or at least with carers. Subjects who could not attend our hospital, such as ones who received intensive nursing care at home or at a nursing house, were not enrolled. In addition, we limited the subjects to the elderly. Comparisons with younger patients will be valuable for a deeper understanding of the prognosis of implants with ISFDPs.

## 5. Conclusions

Within the limitations of this study, the implants and ISFDPs showed a high survival rate in the elderly population for up to 10 years. Peri-implantitis was the major reason for implant loss, suggesting the importance of regular maintenance. In most patients, less- or non-invasive procedures were used after the loss of implants. In some patients, efforts to simplify the oral environment and oral care were employed, as previous studies have recommended. Further studies to assess the effect of these procedures are required.

## Figures and Tables

**Table 1:** Inclusion and exclusion of subjects.

		Patients	Implants	Prostheses
Total number during study period		1220	3808	1866
exclusion	Age < 65 years old	1020	3107	1573
	Observation period < 5 years after the placement of definitive ISFDP	4	13	5
	Removable prostheses were delivered	1	1	1
Total number included in the present study		195	687	287

**Table 2:** Implant distribution.

Site		Anterior			Posterior			Anterior–Posterior		Total
Number of Units		1	2	≥3	1	2	≥3	2	≥3	
Maxilla	IMP	24 (15 ISFDPs)			205 (97 ISFDPs)			110 (21 ISFDPs)		339 (133 ISFDPs)
	Pt	8	2	5	18	45	34	0	21	
Mandible	IMP	9 (5 ISFDPs)			260 (132 ISFDPs)			79 (17 ISFDPs)		348 (154 ISFDPs)
	Pt	2	0	3	27	70	35	1	16	
Total IMPs (Total ISFDPs)		33 (20 ISFDPs)			465 (229 ISFDPs)			189 (38 ISFDPs)		687 (287 ISFDPs)

IMP: implant; Pt: patients; ISFDPs: implant-supported fixed dental prostheses.

**Table 3:** Prosthetic conditions (retention type and materials of the suprastructure).

	Single Unit (55 ISFDPs)	2-Unit (118 ISFDPs)	≥3-Unit (114 ISFDPs)	Total (287 ISFDPs)
Screw-retained	49	111	102	262
Cement-retained	6	7	12	25
Metal	7	12	3	22
Metal–resin	13	47	60	120
Metal–ceramic	30	53	48	131
All ceramic	1	1	2	4
Monolithic zirconia	4	5	1	10

ISFDPs: implant-supported fixed dental prostheses.

**Table 4:** The cumulative survival rates of implants up to 10 years.

Years	Number of Implants	Loss	Cumulative Survival Rate (%)	95% Confidence Interval
0–5	687	7	99.0	97.9–99.5
5–6	680	1	98.8	97.7–99.4
6–7	538	1	98.6	97.4–99.3
7–8	423	2	98.1	96.6–99.0
8–9	279	0	98.1	96.6–99.0
9–10	133	0	98.1	96.6–99.0

**Table 5:** Detailed data of the lost implants.

Age (Baseline) Gender	Period (Months)	Site, Unit, Retention, Material	Reason for Loss	Intervention after Loss
≤5 years				
67 Female	22	46, 2-unit, SC, MC	Discomfort due to implant position	IARPD (implant: 45 + RPD)
65 Male	43	46, 12-unit, SC, MC	Peri-implantitis	Implant placement (implants: 44, 45 + cantilever: 46)
66 Female	46	26, 2-unit, SC, MR	Peri-implantitis	Modification of suprastructure (implant: 25 + SDA)
71 Male	46	46, 2-unit, SC, MR	Peri-implantitis	Implant placement (implant: 46)
67 Female	50	46 and 47, 2-unit, SC, MR	Peri-implantitis	RPD
65 Female	53	16, 4-unit, SC, MC	Peri-implantitis	Modification of suprastructure (pontic: 16)
>5 years				
76 Male	66	35, 3-unit, SC, MR	Bone resection due to gingival cancer	IARPD (implant: 37 + RPD)
75 Male	79	36, Single, SC, MR	Peri-implantitis	RPD
68 Male	88	36, 2-unit, SC, MC	Bone resorption	Modification of suprastructure (implant: 37 + cantilever: 36)
66 Male	89	44, 3-unit, SC, MR	Peri-implantitis	Modification of suprastructure (implants: 45, 46 + cantilever: 44)

SC: screw-retained; MC: metal–ceramic; MR: metal–resin; IARPD: implant-assisted removable partial denture; RPD: removable partial denture; SDA: shortened dental arch.

## Data Availability

The datasets generated during the current study are available from the corresponding author on reasonable request.
